# Incidence of Fit Test Failure During N95 Respirator Reuse and Extended Use

**DOI:** 10.1001/jamanetworkopen.2023.53631

**Published:** 2024-01-26

**Authors:** Ralph C. Wang, Nida F. Degesys, Jahan Fahimi, Chengshi Jin, Efrat Rosenthal, Ann A. Lazar, Anna Q. Yaffee, Susan Peterson, Richard E. Rothmann, Courtney M. C. Jones, Vaishal Tolia, Manish N. Shah, Maria C. Raven

**Affiliations:** 1 Department of Emergency Medicine, University of California, San Francisco; 2 Department of Epidemiology & Biostatistics, University of California, San Francisco; 3 Department of Emergency Medicine, Emory University, Atlanta, Georgia; 4 Department of Emergency Medicine, Johns Hopkins University, Baltimore, Maryland; 5 Department of Emergency Medicine, University of Rochester Medical Center, Rochester, New York; 6 Department of Emergency Medicine, University of California, San Diego; 7 BerbeeWalsh Department of Emergency Medicine, University of Wisconsin, Madison; 8 Philip R. Lee Institute for Health Policy Studies, University of California, San Francisco

## Abstract

**Importance:**

The COVID-19 pandemic resulted in a widespread acute shortage of N95 respirators, prompting the Centers for Disease Control and Prevention to develop guidelines for extended use and limited reuse of N95s for health care workers (HCWs). While HCWs followed these guidelines to conserve N95s, evidence from clinical settings regarding the safety of reuse and extended use is limited.

**Objective:**

To measure the incidence of fit test failure during N95 reuse and compare the incidence between N95 types.

**Design, Setting, and Participants:**

This prospective cohort study, conducted from April 2, 2021, to July 15, 2022, at 6 US emergency departments (EDs), included HCWs who practiced N95 reuse for more than half of their clinical shift. Those who were unwilling to wear an N95 for most of their shift, repeatedly failed baseline fit testing, were pregnant, or had facial hair or jewelry that interfered with the N95 face seal were excluded.

**Exposures:**

Wearing the same N95 for more than half of each clinical shift and for up to 5 consecutive shifts. Participants chose an N95 model available at their institution; models were categorized into 3 types: dome (3M 1860R, 1860S, and 8210), trifold (3M 1870+ and 9205+), and duckbill (Halyard 46727, 46767, and 46827). Participants underwent 2 rounds of testing using a different mask of the same type for each round.

**Main Outcomes and Measures:**

The primary outcome was Occupational Safety and Health Administration–approved qualitative fit test failure. Trained coordinators conducted fit tests after clinical shifts and recorded pass or fail based on participants tasting a bitter solution.

**Results:**

A total of 412 HCWs and 824 N95s were fit tested at baseline; 21 N95s (2.5%) were withdrawn. Participants’ median age was 34.5 years (IQR, 29.5-41.8 years); 252 (61.2%) were female, and 205 (49.8%) were physicians. The overall cumulative incidence of fit failure after 1 shift was 38.7% (95% CI, 35.4%-42.1%), which differed by N95 type: dome, 25.8% (95% CI, 21.2%-30.6%); duckbill, 28.3% (95% CI, 22.2%-34.7%); and trifold, 61.3% (95% CI, 55.3%-67.3%). The risk of fit failure was significantly higher for trifold than dome N95s (adjusted hazard ratio, 1.75; 95% CI, 1.46-2.10).

**Conclusions and Relevance:**

In this cohort study of ED HCWs practicing N95 reuse, fit failure occurred in 38.7% of masks after 1 shift. Trifold N95s had higher incidence of fit failure compared with dome N95s. These results may inform pandemic preparedness, specifically policies related to N95 selection and reuse practices.

## Key Points

**Question:**

What is the incidence of qualitative fit test failure during the extended use and limited reuse of N95 respirators, and does this vary by N95 type?

**Findings:**

In this cohort study of 824 N95s used by 412 emergency department (ED) health care workers (HCWs) practicing N95 extended use and reuse, the overall incidence of fit failure was 38.7% after 1 shift and differed significantly by N95 type: dome, 25.8%; duckbill, 28.3%; and trifold, 61.3%.

**Meaning:**

The findings suggest substantial rates of fit failure among HCWs practicing N95 reuse, which could inform policies related to N95 selection and reuse practices.

## Teaser

This cohort study measures the incidence of qualitative fit test failure of dome, duckbill, and trifold N95 respirators reused by emergency department health care workers during a single shift and over multiple shifts.

## Introduction

Frontline health care workers (HCWs) are at high risk of acquiring respiratory viral infections, including influenza and SARS-CoV-2 infections.^1,2^ During the COVID-19 pandemic, HCWs used personal protective equipment (PPE), such as an N95 filtering facepiece respirator (hereafter, N95), to reduce their risk of becoming infected.^3,4^ N95s are tight-fitting, disposable respirators designed to prevent aerosolized transmission of viral particles and reduce the likelihood of HCWs acquiring a respiratory viral infection, thus mitigating risk for an essential workforce.^4,5,6,7^ Under nonpandemic circumstances, standard practice is to discard an N95 after close contact with a patient (ie, single use). Prior infectious disease outbreaks and pandemics necessitating N95 conservation prompted the Centers for Disease Control and Prevention to revise its recommendation to include guidelines for both extended use (wearing the same N95 for multiple patient encounters without donning and doffing) and limited reuse (storing an N95 between donnings for no more than 5 donnings), albeit with limited evidence.^8,9^ Frontline HCWs (including those in emergency departments [EDs]) generally followed these guidelines to conserve PPE during the COVID-19 pandemic, practicing a combination of reuse and extended use of N95s, which we collectively term *reuse*.^10^ In practice, patterns of use varied across EDs based on a number of factors, including supply chain and local hospital guidance.

Several laboratory-based experimental studies evaluating the safety of reuse found that N95 fit was reduced after 5 donnings and doffings.^11,12,13^ However, evidence of the safety of reuse practices in clinical settings is lacking,^14^ as few prospective studies have been conducted to explicitly determine the incidence of fit failure during N95 reuse.^15,16,17^ A pilot study conducted by some of us in 1 ED found that N95 reuse during increasing numbers of shifts was significantly associated with increased fit test failure.^15^ These studies were limited, as single-center enrollment and cross-sectional designs did not permit definitive estimates of whether N95s could safely be reused.^15,16,17^

We conducted a multicenter prospective cohort study of frontline HCWs across 6 EDs in the US to measure the incidence of qualitative fit test failure during N95 reuse, which included multiple donnings and doffings. We also sought to compare the incidence of fit test failure between HCWs across various N95 types. Our goal was to provide evidence from clinical settings with frontline HCWs to guide N95 reuse policies and conservation efforts, which will have implications for future pandemics.

## Methods

### Study Design and Setting

We conducted a multi-institutional prospective cohort study at 6 EDs in the US from April 2, 2021, to July 15, 2022. Participants were enrolled from the University of California, San Francisco; Emory University; Johns Hopkins University; the University of California, San Diego; the University of Rochester; and the University of Wisconsin–Madison. The institutional review board at each participating site and the Western Institutional Review Board–Copernicus Group institutional review board approved the study protocol. All participants provided written informed consent. We adhered to the Strengthening the Reporting of Observational Studies in Epidemiology (STROBE) reporting guideline.^18^

### Study Participants

We enrolled ED HCWs, including physicians, nurses, advanced practice providers, and other staff who provided direct clinical care for patients. We included HCWs older than 18 years who practiced N95 reuse for more than half of each shift and were scheduled to work a minimum of 10 shifts within a 6-month period. As the hours of enrollment and data collection depended on research associate availability, participants working certain shifts were not eligible. This varied by site, as some sites enrolled 24 hours a day and 7 days a week, whereas other sites enrolled on weekdays between 7 am and 11 pm. We excluded HCWs who were unwilling to wear an N95 for most of their shift, failed baseline fit testing more than 3 times, were pregnant, or had facial hair or jewelry that interfered with the face seal of an N95.^3^ We excluded pregnant participants due to the changes in body weight during pregnancy, which could impact respirator fit.^19^ Participants’ race (Asian, Black or African American, Hawaiian or other Pacific Islander, White, multiracial, or other [Mestizo, Persian, and mixed Hispanic]) and ethnicity (Hispanic or not Hispanic) were ascertained by self-report and were included in the analysis to assess whether enrollment was inclusive and the cohort was racially and ethnically diverse.

### Study Procedures

Participants chose an N95 that was available at their institution. We fit tested participants using a qualitative fit test at baseline and then at the end of their clinical shifts (up to 5 consecutive shifts) with the same N95. A sealable, rigid, nonbreathable plastic container with a lid was provided to participants to store their N95 between shifts. Each participant wore the N95 until fit failure, heavy soiling, or deforming or until 5 shifts had elapsed. During each shift, participants wore their N95 for multiple patients and were asked to limit their donning and doffing to 5 times per shift. Participants underwent 2 rounds of testing using different masks of the same type for each round, thus contributing 2 N95 respirators. Participants received a $100 gift card after study completion. Duration of participant shifts varied from 8 to 12 hours.

### Primary Outcome

The primary outcome of the study was Occupational Safety and Health Administration (OSHA)–approved qualitative fit test failure.^20^ Trained fit testers conducted standardized fit tests at the end of participant clinical shifts (eAppendix in Supplement 1). In brief, a hood was placed over the participant wearing an N95, and FT-32 bitter testing solution (3M) was sprayed while the participant performed several exercises, including breathing, head turns, and walking. Fit failure was defined as the participant tasting the bitter solution at any time during the fit test. While previous studies have used quantitative fit testing,^11,13,21^ we used qualitative fit test failure, as it is the current OSHA procedure used to determine the fit of N95s.^22^ We considered N95 strap breakage as fit failure.^11^ During the fit testing, coordinators did not aid participants in donning or doffing their N95s. Donning and doffing was done at the discretion of the participants.

### Study Exposure and Key Variables

To assess the association between N95 type and fit failure, we surveyed sites to determine the N95 manufacturer and model prior to study initiation. N95s were categorized into 3 types, similar to the pilot study^15^: dome (3M 1860R, 1860S, and 8210), trifold (3M 1870+ and 9205+), and duckbill (Halyard 46727, 46767, and 46827). At the baseline study visit, we recorded participant demographics, job type, characteristics of the N95 (manufacturer, model, lot number, and expiration date), and number of bitter solution sprays needed for the participant to taste the solution. At the end of each shift, we collected data related to N95 use that could impact fit failure (number of donnings and doffings, hours worn, and shift length). We asked participants to provide the number of donnings and doffings that they performed during each shift based on their recall. Participants were provided click counters to help record their donnings and doffings, and at the beginning of the study, they were asked to limit their donning and doffing to 5 times or less per shift. Study staff also recorded variables associated with N95 reuse, including use of skin protectant, makeup, and presence of facial hair or jewelry, after each assessment.^23^

### Statistical Analysis

Descriptive statistics, including medians and ranges for numeric variables and frequencies and percentages for categorical variables, were used to summarize the data. The primary end point was time to fit test failure, defined as the time from the first time the mask was worn until the fit test failure for each mask. We censored N95s if fit test failure did not occur by the end of 5 clinical shifts or if the participant was lost to follow-up. We reported the incidence of qualitative fit test failure by shift and its associated 95% CI. We stratified fit failure by N95 type (dome, duckbill, and trifold) and reported the Gray *P* value, which reduces to the log-rank *P* value for 1 event type.^24^

We used univariable and multivariable Cox proportional hazards regression models to assess the association of potential covariates with fit failure, including gender, age, body mass index, number of donnings and doffings per shift, N95 type, and use of skin protectant.^8,16,22,25^ The pilot study found that the number of shifts worn, number of donnings and doffings, and N95 type were associated with fit failure.^15^ To assess the association between donnings and doffings and fit failure, we used the *t* test to compare the number of donnings and doffings after the first shift in the group that passed the fit test vs those who failed and then subsequently included donnings and doffings per shift in the Cox proportional hazards regression model. We accounted for multiple masks per participant using the marginal Cox proportional hazards regression model approach using the Lin-Wei robust sandwich variance.^26^ The proportional hazards assumption was assessed using an interaction term between each covariate and the log of time. We found that age violated this assumption, and it subsequently was stratified (quartiles of age) in the multivariable Cox proportional hazards regression model via the strata statement in SAS, version 9.4 (SAS Institute Inc). Two-sided *P* < .05 was considered statistically significant. A.A.L. and C. Jin analyzed the data using SAS, version 9.4.

The preliminary pilot study^15^ found an overall fit test failure proportion of 14 of 51 tests (27.5%) after 3 or more shifts. With 396 participants experiencing 109 fit test failures, we could obtain an estimated 95% CI of 23% to 33%. Each site was asked to enroll 66 participants, and each participant was asked to test 2 N95s (for a total of 792), which allowed for failure according to N95 model type as a secondary aim.

## Results

**Figure 1.**
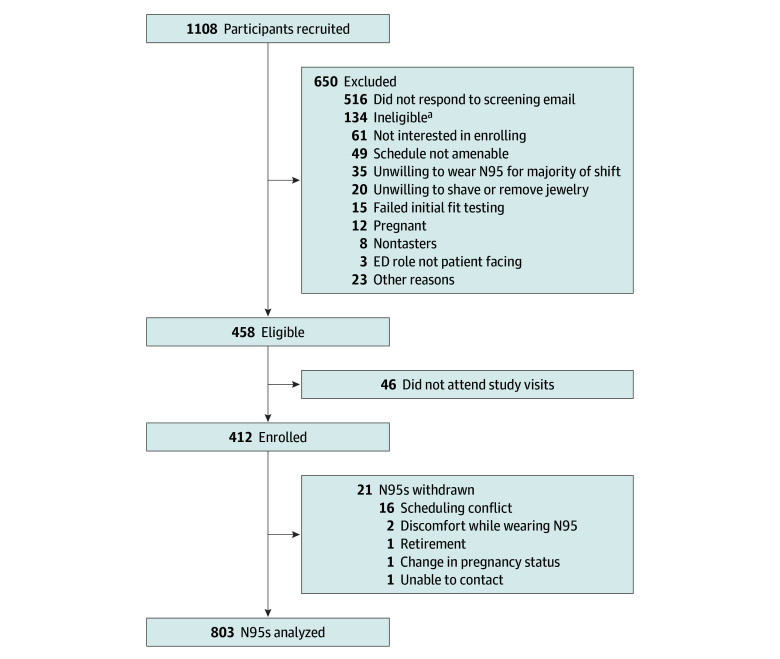
Participant Flow Diagram ^a^More than 1 reason for ineligibility was recorded; 15 participants withdrew 21 N95 respirators from the study.

We recruited 1108 participants from 6 EDs by email. Of the 1108 HCWs invited to participate, 516 did not complete the eligibility screener and 134 were ineligible, resulting in 458 eligible participants (41.3%). A total of 412 participants were enrolled and completed initial fit testing of 2 N95s at baseline (Figure 1). The characteristics of the participants and N95s fit tested at baseline are described in Table 1. Among the 412 participants, the median age was 34.5 years (IQR, 29.5-41.8 years); 252 (61.2%) were female, 158 (38.3%) were male, 1 (0.2%) was nonbinary, and 1 (0.2%) declined to report gender. A total of 55 (13.3%) were Asian; 50 (12.1%), Black or African American; 1 (0.2%), Hawaiian or other Pacific Islander; 33 (8.0%), Hispanic; 375 (91.0%), non-Hispanic; 275 (66.7%), White; 17 (4.1%), multiracial; and 7 (1.7%), other race. Seven (1.7%) declined to report race, and 4 (1.0%) declined to report ethnicity. A total of 205 participants (49.8%) were physicians. Eight different N95 models were chosen and successfully fit tested. The 824 total N95s were categorized into 3 types, including dome (338 [41.0%]), trifold (287 [34.8%]), and duckbill (199 [24.2%]). Twenty-one N95s (2.5%) were withdrawn during follow-up.

**Table 1.** Baseline Characteristics of Study Participants Reusing N95 Respirators

Characteristic	Participants (N = 412)^a^
Age, median (IQR), y	34.5 (29.5-41.8)
Gender	
Female	252 (61.2)
Male	158 (38.3)
Nonbinary	1 (0.2)
Declined to answer	1 (0.2)
Race	
Asian	55 (13.3)
Black or African American	50 (12.1)
Hawaiian or other Pacific Islander	1 (0.2)
White	275 (66.7)
Multiracial	17 (4.1)
Other^b^	7 (1.7)
Declined to answer	7 (1.7)
Ethnicity	
Hispanic	33 (8.0)
Not Hispanic	375 (91.0)
Declined to answer	4 (1.0)
Health care worker type	
Physician	205 (49.8)
Nurse	103 (25)
Nurse practitioner or physician assistant	52 (12.6)
Patient care technician	22 (5.3)
Other	30 (7.3)
Body mass index, median (IQR)^c^	
<18.5	5 (1.2)
18.5-24.9	216 (52.4)
25-29.9	125 (30.3)
≥30	61 (14.8)
Declined to answer	5 (1.2)
Taste threshold	
10 Sprays	376 (91.3)
20 Sprays	29 (7.0)
30 Sprays	7 (1.7)
Facial hair or jewelry	
No	362 (87.9)
Yes	50 (12.1)
N95 manufacturer and model at baseline, No./total No. (%)	
Dome	
3M 1860	175/824 (21.2)
3M 1860 S	131/824 (15.9)
3M 8210	32/824 (3.9)
Trifold	
3M 1870+	248/824 (30.1)
3M 9205+	39/824 (4.7)
Duckbill	
Halyard 46727	118/824 (14.3)
Halyard 46767	29/824 (3.5)
Halyard 46827	52/824 (6.3)

^a^ Data are presented as the number (percentage) of participants unless otherwise indicated.

^b^ Included Mestizo, Persian, and mixed Hispanic.

^c^ Calculated as weight in kilograms divided by height in meters squared.

The characteristics of the N95s used in the first round of the study are described in Table 2; N95s used in the second round are described in eTable 1 in Supplement 1. In round 1, participants wore 405 N95s overall, including 164 dome, 142 trifold, and 99 duckbill N95s. N95 type varied by HCW type. Nurses wore 100 N95s (24.7%) and 47 trifolds (33.1%). Pharmacists and respiratory technicians wore 30 N95s (7.4%) and 20 trifolds (14.1%). N95 type varied by enrolling site. HCWs at sites A and C did not wear trifold N95s, whereas those at site F only used trifolds. The total median number of donnings and doffings did not vary significantly by N95 type, but we identified differences in the median number of donnings and doffings per shift by N95 type (dome, 4 [IQR, 2-6]; trifold, 5 [IQR, 3-10]; duckbill, 4 [IQR, 2-7]). The use of skin protectant varied substantially by N95 type, as skin protectant was used more frequently in the group using dome N95s (33 [20.1%]) compared with the trifold (12 [8.5%]) and duckbill (7 [7.1%]) groups.

**Table 2.** Characteristics of N95 Respirator Use by Emergency Health Care Workers During Round 1 of Testing, by N95 Type

Characteristic	N95 respirators^a^			
	Overall (n = 405)	Dome (n = 164)	Trifold (n = 142)	Duckbill (n = 99)
Professional type				
Physician	202 (49.9)	84 (51.2)	63 (44.4)	55 (55.6)
Nurse	100 (24.7)	35 (21.3)	47 (33.1)	18 (18.2)
Nurse practitioner or physician assistant	51 (12.6)	24 (14.6)	9 (6.3)	18 (18.2)
Pharmacist or respiratory technician	30 (7.4)	9 (5.5)	20 (14.1)	1 (1.0)
Patient care technician	22 (5.4)	12 (7.3)	3 (2.1)	7 (7.1)
Body mass index^b^				
<18.5	5 (1.2)	4 (2.4)	1 (0.7)	0
18.5-24.9	212 (52.3)	80 (48.8)	82 (57.7)	50 (50.5)
25-29.9	123 (30.4)	49 (29.9)	40 (28.2)	34 (34.3)
≥30	61 (15.1)	31 (18.9)	15 (10.6)	15 (15.2)
Missing	4 (1.0)	0	4 (2.8)	0
Site				
A	78 (19.3)	39 (23.8)	0	39 (39.4)
B	68 (16.8)	49 (29.9)	17 (12.0)	2 (2.0)
C	51 (12.6)	15 (9.1)	0	36 (36.4)
D	46 (11.4)	14 (8.5)	32 (22.5)	0
E	96 (23.7)	47 (28.7)	27 (19.0)	22 (22.2)
F	66 (16.3)	0	66 (46.5)	0
Time respirators were worn, median (IQR), h				
Total	19 (12-31)	22 (14-35)	16 (10-23)	22 (14-32)
Per shift	10 (8-12)	9 (8-12)	10 (9-13)	10 (8-12)
Donnings and doffings, median (IQR), No.				
Total	9 (5-17)	10 (4-17)	8 (5-17)	9 (5-15)
Per shift	4 (2-7)	4 (2-6)	5 (3-10)	4 (2-7)
Facial coverings				
Use of makeup	41 (10.1)	17 (10.4)	13 (9.2)	11 (11.1)
Use of skin protectant	52 (12.8)	33 (20.1)	12 (8.5)	7 (7.1)
Facial hair or jewelry	41 (10.1)	22 (13.4)	9 (6.3)	10 (10.1)

^a^ Data are presented as the number (percentage) of N95 respirators unless otherwise indicated.

^b^ Calculated as weight in kilograms divided by height in meters squared.

**Figure 2.**
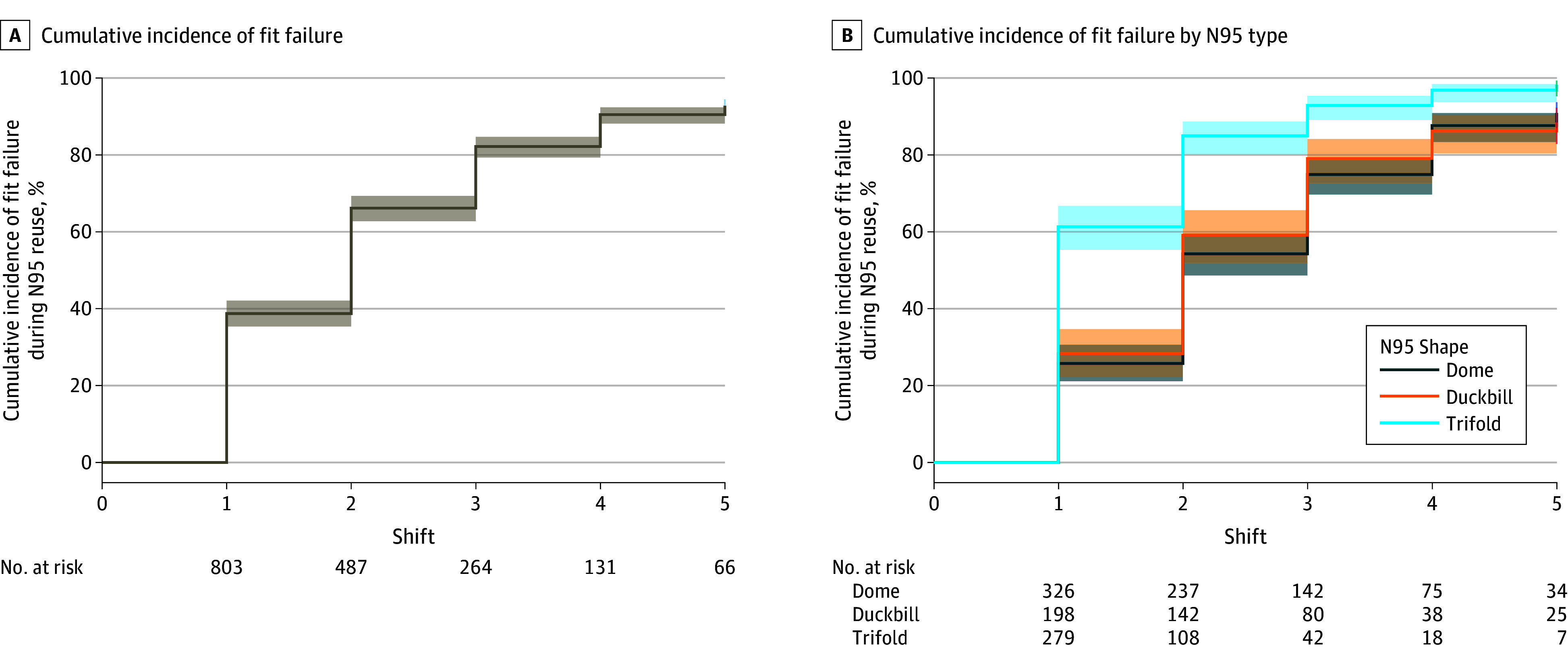
Incidence of N95 Respirator Fit Failure During Reuse Shading indicates 95% CIs.

Figure 2A shows the cumulative incidence of fit failure over 5 shifts. After 1 shift of wear, the overall cumulative incidence of fit failure was 38.7% (95% CI, 35.4%-42.1%). By 5 shifts of wear, 92.8% (95% CI, 90.7%-94.4%) of N95s had failed. Figure 2B depicts the cumulative incidence of fit failure stratified by N95 type. Dome (25.8%; 95% CI, 21.2%-30.6%) and duckbill (28.3%; 95% CI, 22.2%-34.7%) N95s had similar cumulative incidence of failure after 1 shift, whereas trifold N95s had the highest cumulative incidence after 1 shift (61.3%; 95% CI, 55.3%-67.3%). This pattern of fit failure incidence was also observed for individual models (eTable 2 in Supplement 1).

In the Cox proportional hazards regression ratio model, several variables were significantly associated with fit test failure in univariate analyses, including N95 type, donning and doffing, gender, age, and use of skin protectant (Table 3). In a multivariable model controlling for these variables, N95 type was independently associated with fit test failure. Trifold N95s had the highest likelihood of fit failure, with an adjusted hazard ratio (HR) of 1.75 (95% CI, 1.46-2.10) compared with dome type. Female gender was also associated with a higher likelihood of fit failure (adjusted HR, 1.19; 95% CI, 1.01-1.40). Use of skin protectant was associated with a reduced likelihood of fit failure (adjusted HR, 0.74; 95% CI, 0.58-0.94).

**Table 3.** Unadjusted and Adjusted Hazard Ratios for Incidence of N95 Respirator Fit Failure During Reuse

Covariate	Hazard ratio (95% CI)^a^	
	Unadjusted	Adjusted^b^
N95 type		
Dome	1 [Reference]	1 [Reference]
Duckbill	1.03 (0.85-1.24)	0.98 (0.81-1.19)
Trifold	1.80 (1.52-2.14)	1.75 (1.46-2.10)
Gender		
Female	1.10 (0.95-1.28)	1.19 (1.01-1.40)
Male	1 [Reference]	1 [Reference]
Body mass index^c^		
<18.5	0.71 (0.30-1.38)	0.78 (0.33-1.54)
18.5-24.9	1 [Reference]	1 [Reference]
25-29.9	0.94 (0.79-1.11)	1.03 (0.87-1.22)
≥30	0.94 (0.76-1.16)	1.08 (0.87-1.35)
Use of skin protectant	0.79 (0.62-0.98)	0.74 (0.58-0.94)
Donning and doffing per shift	1.02 (1.00-1.03)	1.01 (0.99-1.02)

^a^ Site was included as a fixed effect.

^b^ Adjusted for gender, body mass index, use of skin protectant, and the number of donnings and doffings.

^c^ Calculated as weight in kilograms divided by height in meters squared.

To explore the association between donning and doffing and fit failure, we examined all participants’ mean number of donnings and doffings during shift 1, comparing N95s that failed the fit test after shift 1 with masks that did not. We found that significantly less donning and doffing occurred among those that passed the fit test after the first shift compared with those that failed (mean [SD] donnings and doffings, 5.6 [5.6] vs 7.1 [7.1]; *P* = .002). We included mean donning and doffing per shift in the multivariable model. A greater number of donnings and doffings per shift was associated with fit failure in the univariate proportional hazards model but not after adjustment in the multivariate model.

## Discussion

In this prospective cohort study of N95s worn by HCWs practicing extended N95 use and reuse, we found high rates of fit failure after 1 shift, and 92.8% of N95s failed after 5 shifts. We found differences in fit failure by N95 type. Trifold N95s failed at significantly higher rates compared with dome and duckbill N95s even after adjustment in a multivariate hazard model including gender, donnings and doffings per shift, HCW type, body mass index, and use of skin protectant. While the rate of fit failure varied by N95 type, even dome N95s, with the lowest incidence of fit failure, had high rates of fit failure after 1 shift. These results provide evidence to guide reuse of N95 respirators in clinical settings^14^ and apply to other respiratory virus outbreaks when reuse may be considered within health care settings.

These results differ from those of an initial cross-sectional study by some of us of ED professionals who wore either a 3M 1860 dome style N95 or a duckbill N95.^15^ That pilot study found lower rates of fit failure (7.1%; 95% CI, 0.2%-33.9%) when HCWs reused dome N95s for 1 shift. These differences in findings are likely attributable to the pilot study using a less rigorous design and protocol (including no initial fit testing) and differences in the timing of fit tests. In addition, the pilot study had a small sample size and wide 95% CIs. The failure rates in the current study were also higher than those in another single-center cross-sectional study that found fit failure in 33.3% of masks after 2 shifts and 42.9% after 3 shifts.^16^

When restricting our analysis to only shift 1, fit failure was significantly associated with increased donning and doffing on that shift. A prior nonclinical study found that as donning and doffing increased, fit factors (as measured by the quantitative respirator fit-testing equipment) decreased, with a suggested cutoff of 5 consecutive donnings and doffings.^9^ The longer that participants wore a mask in our study, the more chances they had to don and doff, which could create a false association between increased donning and doffing and lower fit failure risk. We did not find donning and doffing to be associated with fit failure in multivariable analyses.

Our finding of significantly higher fit failure rates among trifold N95s compared with other models has implications for their future reuse. Our findings suggest that reuse of trifold N95s should be avoided. Finally, our results add to concerns that efforts should be made to avoid critical PPE shortages as were experienced during the early COVID-19 pandemic.^27^ Factors that contributed to severe shortages during the COVID-19 crisis included panicked marketplace behavior, lack of effective action to maintain and distribute domestic inventories, and severe disruptions to the PPE global supply chain.^9,28^ As the safety of N95 reuse beyond 1 clinical shift is uncertain, our findings underscore the importance of pandemic preparedness, including ensuring an adequate PPE stockpile, an ability to manufacture PPE quickly on a large scale, and increasing use of potentially more effective respirators.^27,29^ This study found that the protective potential of N95s was reduced with an increase in the number of uses and number of donnings, which is also applicable to respiratory viruses other than SARS-CoV-2. Virus infectivity will vary among different viruses and strains, and thus the infectious dose required to cause infection will vary. In addition, the findings of this study apply not only to health care settings but also to other workplaces, such as construction sites and industrial settings, where N95 respirator reuse may be practiced.

### Limitations

We noted several limitations of this study. The qualitative fit test is subjective, based on participant self-report of tasting the bitter solution. A prior study found the qualitative fit test to be oversensitive, with test failures to be falsely positive 71% of the time when using the bitter solution method.^21^ Thus, an N95 that failed a fit test may still protect the wearer. Quantitative measurement using a device such as the PortaCount respirator fit tester (TSI) may have mitigated measurement error but also would have limited the study’s feasibility. Even with the oversensitivity of the fit test, the failure rates we report are unacceptably high. In addition, the fit test is a surrogate outcome for N95 safety, as we did not measure SARS-CoV-2 infection rates after N95 reuse or other measurements of participant SARS-CoV-2 exposure. This study was not designed to assess whether failed fit tests lead to an increase in SARS-CoV-2 infection and instead was designed to examine the fit of N95s during reuse and extended use in clinical settings, which addressed a key knowledge gap for the field of research on respiratory protection and respiratory protection practices.

We are unable to comment on the incidence of fit failure prior to the end of the first shift, as our fit testing occurred at the end of the first shift. We considered performing fit testing during shifts but decided it would not have been feasible in the ED during a pandemic. Finally, the number of donnings and doffings was recorded at the end of each clinical shift and based on participant self-report and thus is subject to recall bias, which may have reduced our ability to assess the true association between donnings and doffings and fit failure.

## Conclusions

In this cohort study of HCWs practicing N95 respirator reuse, the incidence of fit failure after 1 shift was 38.7%. Trifold N95s were associated with the highest cumulative incidence of fit failure. These results should inform pandemic preparedness, specifically policies related to N95 selection and reuse practices during periods of conservation.

## Notes

Supplement 1. **eTable 1.** Characteristics of N95 Use by Emergency HCWs by N95 Type—Round 2 **eTable 2.** Incidence of Fit Failure by N95 Model **eAppendix.** Bitrex Solution Aerosol Qualitative Fit Test Protocol

Supplement 2.
Nonauthor Collaborators

Supplement 3.
Data Sharing Statement
