# Supplementary material for: Incidence of Fit Test Failure During N95 Respirator Reuse and Extended Use
Source: JAMA Netw Open. 2024 Jan 26;7(1):e2353631. doi: 10.1001/jamanetworkopen.2023.53631 (PMC12282505; doi:10.1001/jamanetworkopen.2023.53631)
Supplement: Supplement 1. — eTable 1. Characteristics of N95 Use by Emergency HCWs by N95 Type—Round 2 eTable 2. Incidence of Fit Failure by N95 Model eAppendix. Bitrex Solution Aerosol Qualitative Fit Test Protocol [file jamanetwopen-e2353631-s001.pdf]

Supplementary Online Content

Wang RC, Degesys NF, Fahimi J, et al; Reuse N95 Group. Incidence of fit-test failure during N95 respirator reuse and extended use. *JAMA Netw Open*. 2024;7(1):e2353631. doi:10.1001/jamanetworkopen.2023.53631

eTable 1. Characteristics of N95 Use by Emergency HCWs by N95 Type—Round 2

eTable 2. Incidence of Fit Failure by N95 Model

eAppendix. Bitrex Solution Aerosol Qualitative Fit Test Protocol

This supplementary material has been provided by the authors to give readers additional information about their work.

© 2024 Wang RC et al. *JAMA Network Open*.

eTable1. Characteristics of N95 use by emergency healthcare workers by N95 type (round 2)

Characteristic	Overall<br>N=398	Dome*<br>N=162	Trifold*<br>N=137	Duckbill*<br>N=99
Provider type				
Physician	198 (49.7%)	82 (50.6%)	60 (43.8%)	56 (56.6%)
Nurse	97 (24.4%)	36 (22.2%)	44 (32.1%)	17 (17.2%)
Nurse Practitioner & Physician Assistant	51 (12.8%)	23 (14.2%)	10 (7.3%)	18 (18.2%)
Other (pharmacist, respiratory technician)	30 (7.5%)	9 (5.6%)	20 (14.6%)	1 (1.0%)
Patient Care Technician	22 (5.5%)	12 (7.4%)	3 (2.2%)	7 (7.1%)
BMI categories				
<18.5	5 (1.3%)	4 (2.5%)	1 (0.7%)	0 (0.0%)
18.5-24.9	207 (52.0%)	77 (47.5%)	79 (57.7%)	51 (51.5%)
25-29.9	121 (30.4%)	49 (30.2%)	39 (28.5%)	33 (33.3%)
30+	61 (15.3%)	32 (19.8%)	14 (10.2%)	15 (15.2%)
missing	4 (1.0%)	0 (0.0%)	4 (2.9%)	0 (0.0%)
Site				
A	78 (19.6%)	39 (24.1%)	0 (0.0%)	39 (39.4%)
B	67 (16.8%)	49 (30.2%)	16 (11.7%)	2 (2.0%)
C	51 (12.8%)	15 (9.3%)	0 (0.0%)	36 (36.4%)
D	43 (10.8%)	13 (8.0%)	30 (21.9%)	0 (0.0%)
E	93 (23.4%)	46 (28.4%)	25 (18.2%)	22 (22.2%)
F	66 (16.6%)	0 (0.0%)	66 (48.2%)	0 (0.0%)
Hours worn				
Total hours worn (Median, IQR)	15.5 (10-26)	18 (11-31)	13 (9-18)	18 (11-30)
Hours worn per shift (Median (IQR)	10 (8-12.5)	9 (7.5-12)	11 (9-13)	9.5 (8-11.7)
Donning and Doffing				
Total Donning and doffings (Median, IQR)	9 (4-15)	8 (4-16)	8 (4-15)	9 (5-14)
Donning and doffings per shift	4.63 (3-8)	4 (2.4-6)	6 (3-10)	4.5 (3-7)
Facial Coverings				
Use of make up	32 (8.0%)	16 (9.9%)	8 (5.8%)	8 (8.1%)
Use of skin protectant	47 (11.8%)	29 (17.9%)	11 (8.0%)	7 (7.1%)
Facial hair or jewelry	38 (9.5%)	19 (11.7%)	10 (7.3%)	9 (9.1%)

[1] Based on Kruskal-Wallis test; [2] Based on Chi-square test

*Dome N95 included the 3M 1860, 1860 S, and 3M 8210 models. Trifold N95s included the 3M 1870 and 3M 9205 models, and duckbill N95s included the Halyard models.

© 2024 Wang RC et al. *JAMA Network Open*.

eTable 2: Incidence of Fit Failure by N95 Model

	Shift 1	Shift 2	Shift 3	Shift 4	Shift 5
Dome	25.8% (21.2-30.6%)	54.3% (48.7-59.5%)	74.9% (69.7-79.3%)	87.6% (83.3-90.9%)	90.9% (87.0-93.7%)
3M 1860	23.1% (17.1-29.7%)	45.7% (38.1-52.9%)	71.4% (64.1-77.5%)	83.9% (77.4-88.7%)	87.9% (81.8-92.1%)
3M 1860S	26.6% (19.2-34.6%)	62.5% (53.0-70.5%)	78.0% (69.1-84.6)	93.0% (85.9-96.6%)	96.0% (89.3-98.5%)
3M 8210	37.9% (20.6-55.2%)	72.4% (51.7-85.4%)	82.8% (62.3-92.7%)	89.7% (69.1-96.8%)	NA
Trifold	61.3% (55.3-66.7%)	85.0% (80.2-88.7%)	92.8% (89.1-95.3%)	96.8% (93.7-98.4%)	98.2% (95.2-99.3%)
3M 1870+	62.9% (56.5-68.9%)	85.0% (79.8-89.0%)	92.5% (88.3-95.2%)	96.7% (93.2-98.4%)	97.8% (94.4-99.2%)
3M 9205+	51.3% (34.6-65.7%)	84.6% (68.4-92.9%)	94.9% (79.2-98.8%)	97.4% (73.6-99.8%)	100% (NA)
Duckbill	28.3% (22.2-34.7%)	59.1% (51.9-65.6%)	79.0% (72.6-84.1%)	86.2% (80.4-90.4%)	88.4% (82.8-92.3%)
Halyard 46727	25.4% (18.0-33.6%)	58.5% (49.0-66.8%)	78.0.8% (69.3-84.5%)	83.7% (75.4-89.4%)	85.6% (77.5-91.0%)
Halyard 46767	39.3% (21.4-56.8%)	75.0% (53.9-87.5%)	83.3% (61.1-93.5%)	91.7% (67.3-98.1%)	NA
Halyard 46827	28.9% (17.2-41.5%)	51.9% (37.5-64.6%)	78.9% (64.8-87.8%)	88.5% (79.8-94.8%)	92.3% (79.8-97.2%)

© 2024 Wang RC et al. *JAMA Network Open*.

eAppendix. Bitrex solution aerosol qualitative fit test protocol

The BitrexTM (Denatonium benzoate) solution aerosol QLFT protocol uses the published saccharin test protocol because that protocol is widely accepted. Bitrex is routinely used as a taste aversion agent in household liquids which children should not be drinking and is endorsed by the American Medical Association, the National Safety Council, and the American Association of Poison Control Centers. The entire screening and testing procedure shall be explained to the test subject prior to the conduct of the screening test.

(a) Taste Threshold Screening. *The Bitrex taste threshold screening, performed without wearing a respirator, is intended to determine whether the individual being tested can detect the taste of Bitrex.*

(1) During threshold screening as well as during fit testing, subjects shall wear an enclosure about the head and shoulders that is approximately 12 inches (30.5 cm) in diameter by 14 inches (35.6 cm) tall. The front portion of the enclosure shall be clear from the respirator and allow free movement of the head when a respirator is worn. An enclosure substantially similar to the 3M hood assembly, parts # FT 14 and # FT 15 combined, is adequate.

(2) The test enclosure shall have a 3⁄4-inch (1.9 cm) hole in front of the test subject's nose and mouth area to accommodate the nebulizer nozzle.

(3) The test subject shall don the test enclosure. Throughout the threshold screening test, the test subject shall breathe through his or her slightly open mouth with tongue extended. The subject is instructed to report when he/she detects a bitter taste.

(4) Using a DeVilbiss Model 40 Inhalation Medication Nebulizer or equivalent, the test conductor shall spray the Threshold Check Solution into the enclosure. This Nebulizer shall be clearly marked to distinguish it from the fit test solution nebulizer.

(5) The Threshold Check Solution is prepared by adding 13.5 milligrams of Bitrex to 100 ml of 5% salt (NaCl) solution in distilled water.

(6) To produce the aerosol, the nebulizer bulb is firmly squeezed so that the bulb collapses completely and is then released and allowed to fully expand.

(7) An initial ten squeezes are repeated rapidly and then the test subject is asked whether the Bitrex can be tasted. If the test subject reports tasting the bitter taste during the ten squeezes, the screening test is completed. The taste threshold is noted as ten regardless of the number of squeezes completed.

(8) If the first response is negative, ten more squeezes are repeated rapidly, and the test subject is again asked whether the Bitrex is tasted. If the test subject reports tasting the bitter taste during the second ten squeezes, the screening test is completed. The taste threshold is noted as twenty regardless of the number of squeezes completed.

© 2024 Wang RC et al. *JAMA Network Open*.

(9) If the second response is negative, ten more squeezes are repeated rapidly, and the test subject is again asked whether the Bitrex is tasted. If the test subject reports tasting the bitter taste during the third set of ten squeezes, the screening test is completed. The taste threshold is noted as thirty regardless of the number of squeezes completed.

(10) The test conductor will take note of the number of squeezes required to solicit a taste response.

(11) If the Bitrex is not tasted after 30 squeezes (step 10), the test subject is unable to taste Bitrex and may not perform the Bitrex fit test.

(12) If a taste response is elicited, the test subject shall be asked to take note of the taste for reference in the fit test.

(13) Correct use of the nebulizer means that approximately 1 ml of liquid is used at a time in the nebulizer body.

(14) The nebulizer shall be thoroughly rinsed in water, shaken to dry, and refilled at least each morning and afternoon or at least every four hours.

(b) Bitrex Solution Aerosol Fit Test Procedure.

(1) The test subject may not eat, drink (except plain water), smoke, or chew gum for 15 minutes before the test.

(2) The fit test uses the same enclosure as that described in 4. (a) above.

(3) The test subject shall don the enclosure while wearing the respirator selected according to section I. A. of this appendix. The respirator shall be properly adjusted and equipped with any type particulate filter(s).

(4) A second DeVilbiss Model 40 Inhalation Medication Nebulizer or equivalent is used to spray the fit test solution into the enclosure. This nebulizer shall be clearly marked to distinguish it from the screening test solution nebulizer.

(5) The fit test solution is prepared by adding 337.5 mg of Bitrex to 200 ml of a 5% salt (NaCl) solution in warm water.

(6) As before, the test subject shall breathe through his or her slightly open mouth with tongue extended and be instructed to report if he/she tastes the bitter taste of Bitrex.

(7) The nebulizer is inserted into the hole in the front of the enclosure and an initial concentration of the fit test solution is sprayed into the enclosure using the same number of squeezes (either 10, 20 or 30 squeezes) based on the number of squeezes required to elicit a taste response as noted during the screening test.

(8) After generating the aerosol, the test subject shall be instructed to perform the exercises in section I. A. 14. of this appendix.

© 2024 Wang RC et al. *JAMA Network Open*.

(9) Every 30 seconds the aerosol concentration shall be replenished using one half the number of squeezes used initially (e.g., 5, 10 or 15).

(10) The test subject shall indicate to the test conductor if at any time during the fit test the taste of Bitrex is detected. If the test subject does not report tasting the Bitrex, the test is passed.

(11) If the taste of Bitrex is detected, the fit is deemed unsatisfactory and the test is failed. A different respirator shall be tried, and the entire test procedure is repeated (taste threshold screening and fit testing).

© 2024 Wang RC et al. *JAMA Network Open*.
