# Supplementary material for: Incidence of Fit Test Failure During N95 Respirator Reuse and Extended Use
Source: JAMA Netw Open. 2024 Jan 26;7(1):e2353631. doi: 10.1001/jamanetworkopen.2023.53631 (PMC12282505; doi:10.1001/jamanetworkopen.2023.53631)
Supplement: Supplement 2. — Nonauthor Collaborators [file jamanetwopen-e2353631-s002.pdf]

Supplemental Online Content: Nonauthor Collaborators
*First name, last name, and suffix (if applicable) are required and will appear in PubMed.

***Group Name(s): ReUseN95 Group**

***First Name and Middle Initial(s)**	***Last Name**	***Suffix (eg, Jr, III)**	Academic Degrees	Institution	Location (city, state/province, country)	Role or Contribution, eg, chair, principal investigator	Group (if more than 1 Group listed in the byline) and/or Subgroup (eg, Steering Committee)
Anna R.	Harris		MA	Department of Emergency Medicine, University of Cal	San Francisco, CA	Project Manager, Training, Data Collection Tool Build, Data Collection and Cleaning, Editing Manuscript	ReUseN95 Group
Robin	Kemball		MPH	Department of Emergency Medicine, University of Cal	San Francisco, CA	Research Manager	ReUseN95 Group
Alexis	Mitchner		BA	Department of Emergency Medicine, University of Cal	San Francisco, CA	Data Collection, N95 Shipment	ReUseN95 Group
Angela	Wong		BA	Department of Emergency Medicine, University of Cal	San Francisco, CA	Data Collection, N95 Shipment	ReUseN95 Group
Alex	Hall		DHSc, MS, RN	Department of Emergency Medicine, Emory Universit	Atlanta, GA	Co-Investigator	ReUseN95 Group
Rabbiya	Iqbal		BS	Department of Emergency Medicine, Johns Hopkins U	Baltimore, MD	Data Collection, N95 Shipment	ReUseN95 Group
Michael	Kramer		BS	Department of Emergency Medicine, Johns Hopkins U	Baltimore, MD	Data Collection, N95 Shipment	ReUseN95 Group
Kendall	Maliszewski		BS	Department of Emergency Medicine, Johns Hopkins U	Baltimore, MD	Data Collection, N95 Shipment	ReUseN95 Group
Breana	McBryde		BS	Department of Emergency Medicine, Johns Hopkins U	Baltimore, MD	Data Collection, N95 Shipment	ReUseN95 Group
John	DeAngelis		MD	Department of Emergency Medicine, University of Ro	Rochester, NY	Co-Investigator, Data Collection, N95 Shipment	ReUseN95 Group
Emily	Corbett-Valade		MS	Department of Emergency Medicine, University of Ro	Rochester, NY	Data Collection, N95 Shipment	ReUseN95 Group
Edward	Castillo		PhD, MPH	Department of Emergency Medicine, University of Cal	San Diego, CA	Co-Investigator	ReUseN95 Group
Apoorva	Maru		BS, BA	BerbeeWalsh Department of Emergency Medicine, Ur	Madison, WI	Data Collection, N95 Shipment	ReUseN95 Group
Angela	Gifford		MA	BerbeeWalsh Department of Emergency Medicine, Ur	Madison, WI	Data Collection, N95 Shipment	ReUseN95 Group
