# Supplementary material for: Incidence of Fit Test Failure During N95 Respirator Reuse and Extended Use
Source: JAMA Netw Open. 2024 Jan 26;7(1):e2353631. doi: 10.1001/jamanetworkopen.2023.53631 (PMC12282505; doi:10.1001/jamanetworkopen.2023.53631)
Supplement: Supplement 3. — Data Sharing Statement [file jamanetwopen-e2353631-s003.pdf]

Data Sharing Statement

Wang. Incidence of Fit Test Failure During N95 Respirator Reuse and Extended Use. *JAMA Netw Open*. Published January 26, 2024. doi:10.1001/jamanetworkopen.2023.53631

Data
Data available: Yes
Data types: Deidentified participant data
How to access data: Ralph.wang@ucsf.edu
When available: With publication

Supporting Documents
Document types: Informed consent form
How to access documents: Ralph.wang@ucsf.edu
When available: With publication

Additional Information
Who can access the data: researchers whose proposed use of the data has been approved
Types of analyses: for any purpose
Mechanisms of data availability: with a signed data access agreement
